# Identifying the severity of diabetic retinopathy by visual function measures using both traditional statistical methods and interpretable machine learning: a cross-sectional study

**DOI:** 10.1007/s00125-023-06005-3

**Published:** 2023-09-19

**Authors:** David M. Wright, Usha Chakravarthy, Radha Das, Katie W. Graham, Timos T. Naskas, Jennifer Perais, Frank Kee, Tunde Peto, Ruth E. Hogg

**Affiliations:** 1 Centre for Public Health, Queen’s University Belfast, Belfast, UK; 2 Wellcome Wolfson Institute for Experimental Medicine, Queen’s University Belfast, Belfast, UK

**Keywords:** Acuity, Contrast sensitivity, Diabetic retinopathy, Low-luminance acuity, Machine learning, Microperimetry, Perimetry, Statistics, Visual function

## Abstract

**Aims/hypothesis:**

To determine the extent to which diabetic retinopathy severity stage may be classified using machine learning (ML) and commonly used clinical measures of visual function together with age and sex.

**Methods:**

We measured the visual function of 1901 eyes from 1032 participants in the Northern Ireland Sensory Ageing Study, deriving 12 variables from nine visual function tests. Missing values were imputed using chained equations. Participants were divided into four groups using clinical measures and grading of ophthalmic images: no diabetes mellitus (no DM), diabetes but no diabetic retinopathy (DM no DR), diabetic retinopathy without diabetic macular oedema (DR no DMO) and diabetic retinopathy with DMO (DR with DMO). Ensemble ML models were fitted to classify group membership for three tasks, distinguishing (A) the DM no DR group from the no DM group; (B) the DR no DMO group from the DM no DR group; and (C) the DR with DMO group from the DR no DMO group. More conventional multiple logistic regression models were also fitted for comparison. An interpretable ML technique was used to rank the contribution of visual function variables to predictions and to disentangle associations between diabetic eye disease and visual function from artefacts of the data collection process.

**Results:**

The performance of the ensemble ML models was good across all three classification tasks, with accuracies of 0.92, 1.00 and 0.84, respectively, for tasks A–C, substantially exceeding the accuracies for logistic regression (0.84, 0.61 and 0.80, respectively). Reading index was highly ranked for tasks A and B, whereas near visual acuity and Moorfields chart acuity were important for task C. Microperimetry variables ranked highly for all three tasks, but this was partly due to a data artefact (a large proportion of missing values).

**Conclusions/interpretation:**

Ensemble ML models predicted status of diabetic eye disease with high accuracy using just age, sex and measures of visual function. Interpretable ML methods enabled us to identify profiles of visual function associated with different stages of diabetic eye disease, and to disentangle associations from artefacts of the data collection process. Together, these two techniques have great potential for developing prediction models using untidy real-world clinical data.

**Graphical Abstract:**

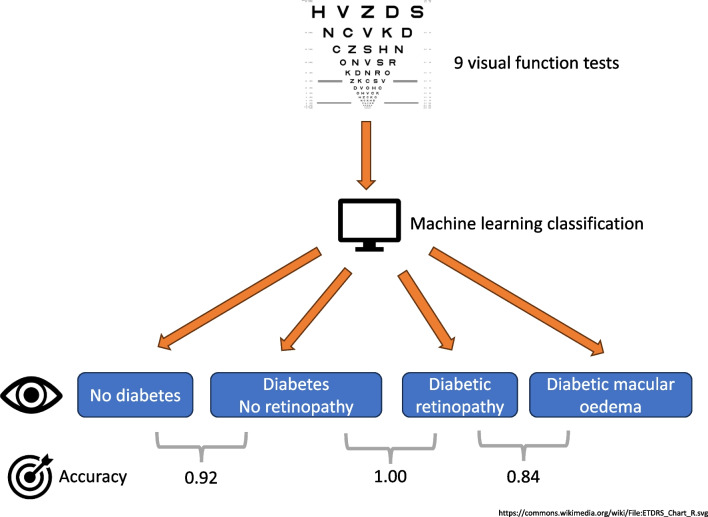

**Supplementary Information:**

The online version of this article (10.1007/s00125-023-06005-3) contains peer-reviewed but unedited supplementary material.


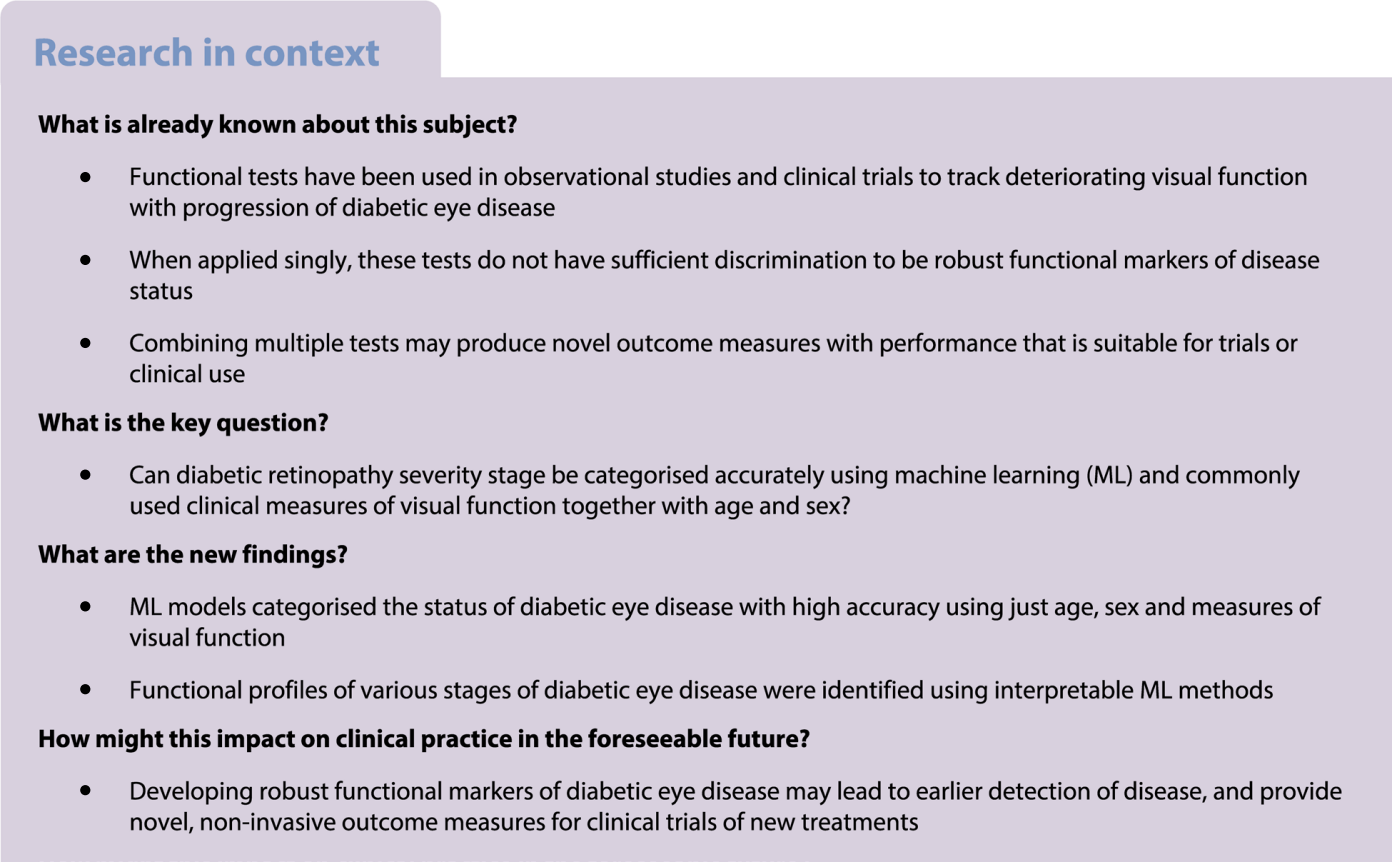


## Introduction

Diabetes mellitus is a major cause of vision loss through development of diabetic retinopathy and diabetic macular oedema (DMO) [1]. Anatomical signs of diabetic retinopathy may take years to develop, but early detection and treatment are crucial to prevent vision loss [2]. While diabetic retinopathy is often described primarily in terms of vascular dysfunction, it has been recognised that the disturbed metabolic environment has a profound impact on neural cells [3]. Many studies now suggest that neural dysfunction precedes the visible vascular signs typically used to diagnose the onset of diabetic retinopathy [4, 5].

Psychophysical tests have been used in observational studies and clinical trials to track deteriorating function with disease progression. However, the structural and spatial heterogeneity of diabetic retinopathy lesions makes identification of direct structure–function relationships challenging when compared with conditions such as glaucoma, in which mathematical models have been found to be useful [6]. Hence, in diabetic retinopathy, less progress has been made in the adoption of robust functional outcomes beyond best corrected distance visual acuity (DVA). However, measurements of DVA can under-represent the visual dysfunction present in diabetes [7], with observational studies suggesting that contrast sensitivity [8], microperimetry [9], dark adaptation [10], matrix perimetry [11] and colour vision [12] are more sensitive outcome measures. However, studies have tended to be small, particularly with respect to the control population used, which is problematic given the inherent noise in psychophysical assessments. Furthermore, they are of insufficient size to account for age effects, and compare few tests, assessing them singly. The heterogeneity of diabetic retinopathy may mean that combinations of tests are more sensitive.

The aim of this study was to determine whether interpretable machine learning (ML) applied across a broad range of visual function measures could distinguish people without diabetes mellitus (no DM group), those who had diabetes mellitus but without overt diabetic retinopathy (DM no DR group), those who had DR but no DMO (DR no DMO group) and those who had diabetic retinopathy with DMO (DR with DMO group).

Three classification tasks were performed, reflecting clinical situations in which determining diabetic retinopathy status rapidly using non-invasive methods would be useful:

(A) distinguishing the retinae of those in the DM no DR group from retinae of normal control individuals (no DM group), mimicking pre-clinical monitoring to determine whether early-stage diabetes changes are detectable in the general population by measuring visual function, which is important because a large proportion of diabetes cases are undiagnosed (41% in our study population [13]).
(B) distinguishing those with diabetic retinopathy but no DMO (DR no DMO group) from those with diabetes mellitus and no diabetic retinopathy (DM no DR group) to evaluate whether clinically relevant diabetic retinopathy features (associated with worse long-term outcomes) are detectable within a diabetes population by measuring visual function.
(C) distinguishing those with DMO (DR with DMO group) from those with diabetic retinopathy without DMO (DR no DMO group), reflecting clinical monitoring of patients with diabetic retinopathy to determine whether sight-threatening DMO is detectable by measuring visual function.

## Methods

### Data collection

Most participants (*n*=881) were part of a prospective population-based epidemiological study of people over 50 years old (Northern Ireland Cohort for the Longitudinal Study of Ageing – NICOLA) [13]. The overall NICOLA study is representative of the Northern Ireland population, but of those that attended the clinical health assessment and were eligible for this study, a greater proportion were in younger age categories, male, retired, had higher levels of education and self-reported health [14]. Participation was higher among those within urban and less-deprived areas. Participants from the following categories were recalled for an additional study visit: those with diabetes mellitus (either self-reported or HbA_1c_ ≥ 48 mmol/mol (6.5%)), those with age-related macular degeneration, and those with no retinal diseases and no diabetes mellitus. Additional participants with confirmed diagnoses of diabetes were recruited from diabetes clinics (*n*=150), together with healthy volunteers (*n*=91) with no history of eye disease aged under 50. These controls were a convenience sample with a similar age distribution to the younger clinical participants, and comprised university employees, their friends or family, or participants recruited via advertising. These groups constitute the Northern Ireland Sensory Ageing (NISA) study (https://clinicaltrials.gov/ct2/show/NCT02788695), comprising 2244 eyes measured across 1122 participants recruited between 2014 and 2018. Participants gave informed consent before taking part and the study was approved by the School of Medicine, Dentistry and Biomedical Sciences Ethics Committee, Queen’s University Belfast, UK (Ref: 12/23, Ref 16.37v2).

We concentrated on diabetic eye disease, excluding 343 eyes with other conditions affecting visual function (including 242 with intermediate/late-stage age-related macular degeneration). Thus, the analysis cohort comprised 1901 eyes from 1032 individuals, 61% of which were from women. The median age of the cohort was 64 years. Half of the eyes from diabetes patients were from NICOLA, and half were from diabetes clinics (see electronic supplementary material [ESM] Table 1). Participants underwent retinal imaging including a fundus colour picture (obtained using a CX-1 digital fundus camera; Canon, USA), colour ultra-wide-field imaging images centred on the fovea (obtained using an Optomap Panoramic 200Tx scanning laser ophthalmoscope; Optos, UK), a macular volume scan (obtained using a Spectralis spectral domain-optical coherence tomograph [SD-OCT]; Heidelberg Engineering, Germany). The volume scan comprised 61 horizontal B-scans (automatic real time [ART] 9) covering a 30×25 degrees rectangle. Imaging and perimetry were performed after pharmacological dilation using 1% tropicamide.

Nine visual function tests were performed (after full refraction) by an experienced optometrist. For perimetry-based tests, the eye with better best corrected DVA was selected for the study, choosing at random if both were eligible [15]. Tests were chosen to cover the breadth of functional deficits previously reported in diabetes, with an emphasis on methodologies that could be easily applied in a clinical setting if found to be predictive.

#### Distance visual acuity

Monocular DVA was evaluated using Early Treatment for Diabetic Retinopathy Study (EDTRS) charts in a light box (Precision Vision, USA) at 4 m, and the total number of letters read was recorded. Best corrected DVA was determined at 4 m distance with all room lights switched off.

#### Near visual acuity

Near visual acuity (NVA) was measured monocularly using Bailey–Lovie near word reading charts at 25 cm with the appropriate reading addition worn over the protocol refraction at 4 m [16] with room lights on. A log minimum angle of resolution (logMAR) score for the smallest line with three or more consecutive words read correctly was recorded.

#### Reading

Reading speed was assessed after the threshold NVA was established. It was tested monocularly using two sets of modified Bailey–Lovie reading speed charts presented as transparencies with black text and corresponding reading speed score sheets [17]. The chart was selected to exhibit text of a print size that was two logarithmic steps larger than the participant’s threshold NVA in the tested eye. The reading index obtained is the reading speed (words per min) divided by the size of print read, thus providing an adjustment for the range of print sizes used [18].

#### Distance low-luminance visual acuity

To measure distance low-luminance visual acuity at 4 m, a 2.0 log neutral-density trial lens was inserted over the final distance refraction result [19, 20]. The low-luminance deficit at distance was the difference in number of letters read between high- and low-luminance best corrected DVA.

#### Near low-luminance visual acuity

To measure near low-luminance visual acuity at 25 cm, the Smith-Kettlewell Institute low-luminance (SKILL) card was used, which measures spatial vision under reduced contrast and luminance [21]. The SKILL card consists of two near acuity charts mounted back-to-back. One has black letters on a dark grey background, simulating reduced contrast and luminance. The other is a high-contrast, black-on-white letter chart. The near low-luminance deficit at near is the acuity loss (number of letters read) between the light and dark sides. The number of letters read from each card (held approximately 40 cm from the patient’s eye wearing the appropriate reading addition) was recorded, with both sides of the chart presented to the participant under normal room lighting.

#### Contrast sensitivity

The contrast sensitivity was measured monocularly using Pelli–Robson charts (Clement Clarke International, UK) viewed at 1 m [22]. A +1.00 dioptre addition trial lens was added to the participant’s distance refractive correction. For a triplet of letters to be scored as ‘seen’, two out of three letters must be correctly identified. Care was taken to ensure uniform illumination of the chart, with luminance from 645–1292 Lux, and to ensure that the chart was concealed from viewing until the test was performed.

#### Moorfields acuity

The Moorfields acuity chart is a distance letter chart comprising vanishing optotypes that have pseudo high-pass design [23]. The mean luminance of the optotypes is the same as the background, so the letters appear to ‘vanish’ soon after the resolution threshold is reached. Such charts are thought to be more robust to optical defocus than traditional letter charts [24]. Visual acuity was measured with the chart under full room illumination (353.8 Lux). For examination of the right eye, we used Moorfields acuity chart 1, while for the left eye, we used Moorfields acuity chart 2.

#### Frequency doubling technology perimetry

The central visual field was assessed on a frequency doubling technology (FDT) Matrix perimeter (Carl Zeiss Meditec, USA) using the 24–2 threshold test. Participants completed the supra-threshold test first to familiarise themselves with the task before undertaking the full threshold test.

#### Microperimetry

Macular integrity assessment was performed using an MAIA macular integrity assessment system (CenterVue, Italy) with a central red circular fixation target and Goldman III stimuli against a background of 1.27 cd/m^2^ using a 4–2 threshold strategy. The maximum stimulus luminance was 318 cd/m^2^, creating a dynamic range of 36 dB. A 45-point customised stimulus grid covering the central 18 degrees was used [25], designed for relatively regular sampling throughout the region, but with slightly increased density towards the fovea.

**Fig. 1:**
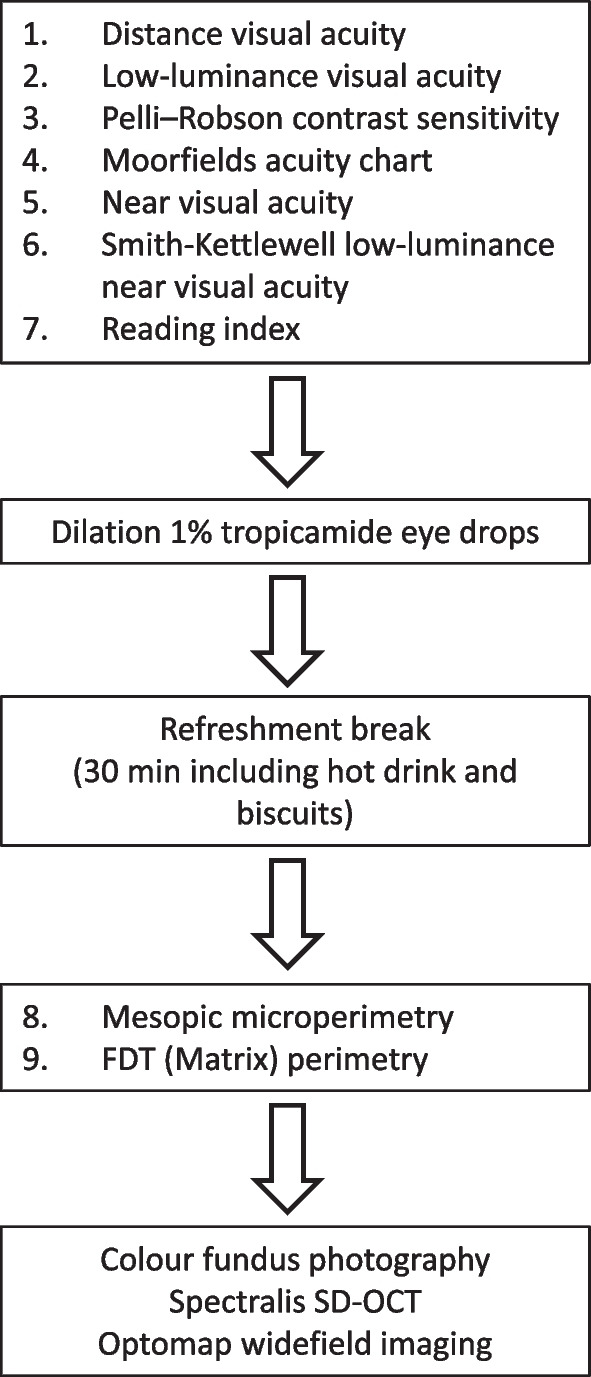
Study visit sequence

Visual function tests were performed in the same sequence for all participants, in two batches with a refreshment break (30 min including hot drink and biscuits) in between to reduce risk of participant fatigue (Fig. 1). Some tests generated multiple variables (e.g. mean deviation and pattern standard deviation from FDT perimetry), giving a total of 12 visual function variables in the analysis dataset, in addition to age and sex. Fundus colour pictures were double graded (by authors RD and UC) to identify signs of age-related macular degeneration (Beckman classification) and diabetic retinopathy. Disc and macula colour images and images obtained by ultra-wide-field imaging were assessed for features of diabetic retinopathy in the central and peripheral retina, and then staged using the national screening for diabetic retinopathy system for England and Wales into four levels: none (R0), background (R1), pre-proliferative (R2) and proliferative (R3) [26]. Participants who were not recruited from the diabetic retinopathy clinic were identified as having diabetes if they self-reported a diabetes diagnosis. Diabetes duration was recorded when provided. Participants over 50 years of age and all patients with diabetes were invited for blood sampling to measure plasma HbA_1c_. Participants with no record of diabetes were classified as having diabetes if their HbA_1c_ was ≥ 48 mmol/mol (6.5%).

### Data preparation

Some visual function variables had a high proportion of missing values (up to 69% for FDT perimetry measures). Only 16.8% of participants had complete information for all variables so imputation was essential to make full use of the dataset. Missing values were imputed using chained equations [27], by imputing missing values for a variable and then using these to impute the next set of missing values until dataset completion. This approach is frequently used to produce multiple imputations (versions) of the input dataset, which are then analysed separately using statistical estimates that are combined using Rubin’s rules. However, this is computationally expensive, and, for this analysis, where the emphasis is on classification rather than estimation, it is unclear how classifications from each version should be combined. Therefore, multiple imputations (six imputed versions for 20 iterations each) were produced for the sole purpose of assessing whether the imputation algorithm had converged on an acceptable solution. A single imputed version was input to the ML pipeline. Numeric variables were normalised prior to model fitting.

### Classification using visual function

One approach to classification is to define ‘normal limits’ (e.g. 2.5% and 97.5% percentiles in a healthy population) and classify measurements outside these limits as abnormal (an approach that is used in perimetry). Previous studies have indicated subtle differences in mean values for some visual function measures when comparing diabetic retinopathy patients with other groups [10, 11]. However, visual function measures have high inter-individual variability, so a classifier constructed using closely overlapping frequency distributions of diabetic retinopathy/DMO and ‘normal’ eyes is likely to perform poorly. Combining multiple variables assessing various aspects of visual function is more powerful, but there is no consensus as to how variables should be combined for any of the three classification tasks. In particular, the ideal functional forms of the relationships between visual function variables and diabetes mellitus/diabetic retinopathy/DMO status are unknown. For example, does the probability of an eye having DMO increase linearly with a decrease in NVA, or is the relationship curvilinear or stepped, perhaps complicated further by interaction with another variable?

We selected a flexible ML approach to capture these complexities, with classifications generated by an ensemble of statistical models (learners) fitted to the same data and combined to produce the best possible classification for each eye in each classification task. The library of learners comprised simple intercept-only models, regression-based models for correlated variables (ridge regression), variable selection (least absolute shrinkage and selection operator [LASSO]) and curvilinear relationships (polynomial splines). In addition, the approach included single-layer neural networks and tree-based methods (XGBoost, random forest and Bayesian additive regression trees) that are capable of modelling threshold-type associations and interactions among multiple variables. For each task, each learner was fitted to 90% of the dataset and used to infer classifications for the remaining 10% (validation set). To avoid overfitting, this was repeated ten times, combining validation sets to give a complete set of classifications (i.e. 10-fold cross-validation [28]). We used individuals as the units of assignment for cross-validation to prevent data leakage if one eye was used to train and the other eye to validate. A final classification for each eye was produced from a linear combination of the learner predictions, fitted using a second stage of ML (a meta-learner). The weights in the linear combination estimated for each learner indicated the relative contribution to the final prediction. The overall algorithm (SuperLearner) achieves the best possible classification provided that one of the learners in the library approximates the true data-generating mechanism [28, 29]. For comparison with a more conventional approach, we also fitted a simpler, main-effects logistic regression model for each task.

Input variables for each task comprised the 12 visual function variables together with age and sex. The output of both the ensemble and logistic models for each eye was the predicted probability of membership of the comparison class for that task (i.e. *P*_DM no DR_, *P*_DR no DMO_ and *P*_DR with DMO_ for tasks A–C, respectively). Eyes with probability >0.5 were labelled into the comparison class; others were labelled into the reference class (no DM, DM no DR and DR no DMO, respectively). Model accuracy was assessed as the proportion of eyes correctly classified. This was compared with the proportion of eyes that would have been labelled correctly if all eyes had been labelled as belonging to the modal class (baseline accuracy). AUC, the area under the receiver operating characteristics (ROC) curve, was calculated as an alternative measure of model performance, which is appropriate for the mild to moderate levels of class imbalance in this dataset (task A, 17% minority class; task B, minority class 44%; class C, minority class 29%).

### Interpretable ML

The ensemble algorithm predicted the probability for each eye, but these probability predictions cannot be directly decomposed as there are too many estimated variables to examine manually and some of the learners are fitted stochastically (e.g. random forests). We used an interpretable ML technique, generating SHapley Additive exPlanation (SHAP) values, to evaluate the contribution of each input variable towards each model prediction [30]. The SHAP technique uses concepts from game theory to define a ‘Shapley value’ for a feature, which provides a measurement of its influence on the underlying model’s prediction. Broadly, this value is calculated for a feature by averaging its marginal contribution to every possible prediction for the instance under consideration. The strategy is straightforward, whereby the technique calculates the marginal contribution of the relevant feature for all possible combinations of inputs in the feature space of the instance. SHAP values may be used to examine the statistical (but not necessarily causal) reasons behind a model prediction, including assessing the importance of different variables and highlighting where predictions depend on statistical artefacts such as missing data. SHAP values are calculated at the level of the prediction (i.e. eye), and so both global and local measures of variable importance may be calculated. Variables associated with greater global variation in SHAP values have greater contribution to probability predictions across the entire dataset. We inspected the profiles of SHAP values for individual eyes to better understand the patterns of model predictions. We also performed clustering by SHAP values to identify clusters of eyes in which predictions were made for similar reasons (see ESM section on interpretable machine learning and ESM Figs 1 and 2). Ensemble models were fitted using the *sl3* package, and SHAP values were calculated using *fastshap* in R version 4.2.1 [31–33].

## Results

### Classification using visual function

Distribution of visual function measures by diabetes and retinopathy status is shown in Table 1. The performance of the ensemble ML models was good, and substantially exceeded that of logistic regression for all three classification tasks (Table 2). Ensemble models exceeded baseline for all tasks, achieving accuracies of 0.92, 1.00 and 0.84 for tasks A–C, respectively, whereas logistic regression exceeded baseline (i.e. confidence intervals did not include baseline) only when distinguishing the DR with DMO group from the DR no DMO group (task C). In terms of the AUC, logistic regression showed moderate performance for task C but poor performance for the other tasks. Ensemble ML models achieved AUC of 1.00 for tasks A and B and 0.93 for task C. The majority of misclassifications were made in the direction of lower disease severity (ESM Table 2).

**Table 1:** Distribution of cohort characteristics and visual function measures by diabetes and retinopathy status

Variable	No DM	DM no DR	DR no DMO	DR with DMO
Eyes	1317	278	216	90
Age (years)	63 (56–69)	67 (60–73)	66 (59–72)	62 (58–67)
Sex				
Female	731 (55.5)	189 (68.0)	167 (77.3)	68 (75.6)
Male	586 (44.5)	89 (32.0)	49 (22.7)	22 (24.4)
Best corrected DVA at 4 m (no. of letters)	85.7±6.71	82.6±11.6	83.5±7.35	73.9±12.4
LLVA at 4 m (no. of letters)	72.9±7.63	69.8±11.8	70.2±8.12	59.1±16.4
Moorfields chart acuity at 4 m (LogMAR)	35.7±6.81	33.1±8.34	34±7.12	22.8±11.3
Pelli–Robson contrast sensitivity (log contrast sensitivity)	1.56±0.174	1.51±0.206	1.48±0.142	1.35±0.231
NVA^a^ (M units)	0.16±0.128	0.21±0.192	0.20±0.14	0.41±0.273
Smith-Kettlewell low-luminance NVA^a^ (no. of letters)	32.3±7.63	33.4±8.67	35.2±8.77	39.0±12.2
Reading index	45.1±9.53	40.0±10.2	37.6±10.9	32.8±13.7
Matrix perimetry mean deviation (dB)	−1.90±3.18	−3.87±4.61	−4.00±4.18	−4.61±3.96
Matrix perimetry pattern standard deviation^a^ (dB)	3.24±0.957	4.04±1.93	4.19±1.5	4.52±1.57
Microperimetry average sensitivity (dB)	26.0±2.52	25.9±2.25	25.3±2.72	22.8±3.16
Microperimetry fixation area 95%^a^ (deg^2^)	4.64±6.83	4.98±7.81	7.62±11	8.01±8.82
Microperimetry central 5-point mean sensitivity (dB)	26.8±2.71	26.3±2.53	25.6±3.27	20.8±5.44

Values are median (IQR), *n* (%) or mean±SD

^a^Low values are better for these variables

LLVA, low-luminance visual acuity; MAR, minimum angle of resolution

**Table 2:** Performance of classification models of diabetes and retinopathy status

Task	Accuracy (baseline)	GLM accuracy	Ensemble accuracy	GLM AUC	Ensemble AUC
A: DM no DR vs no DM	0.83	0.84 (0.82, 0.85)	0.92 (0.91, 0.94)	0.68 (0.65, 0.72)	1.00 (1.00, 1.00)
B: DR no DMO vs DM no DR	0.56	0.61 (0.56, 0.65)	1.00 (0.99, 1.00)	0.64 (0.59, 0.69)	1.00 (1.00, 1.00)
C: DR with DMO vs DR no DMO	0.71	0.80 (0.75, 0.85)	0.84 (0.79, 0.88)	0.86 (0.82, 0.91)	0.93 (0.90, 0.96)

Values are estimates and 95% confidence intervals

Accuracy, proportion of cases classified correctly; GLM, generalised linear model (logistic regression)

The weighting of each component learner towards the ensemble ML predictions varied by classification task (ESM Table 3). Intercept-only models and neural networks were largely disregarded. Random forests contributed most strongly to tasks A and B, and LASSO and Bayesian additive regression trees were highly weighted in task C.

### Interpretable ML

**Fig. 2:**
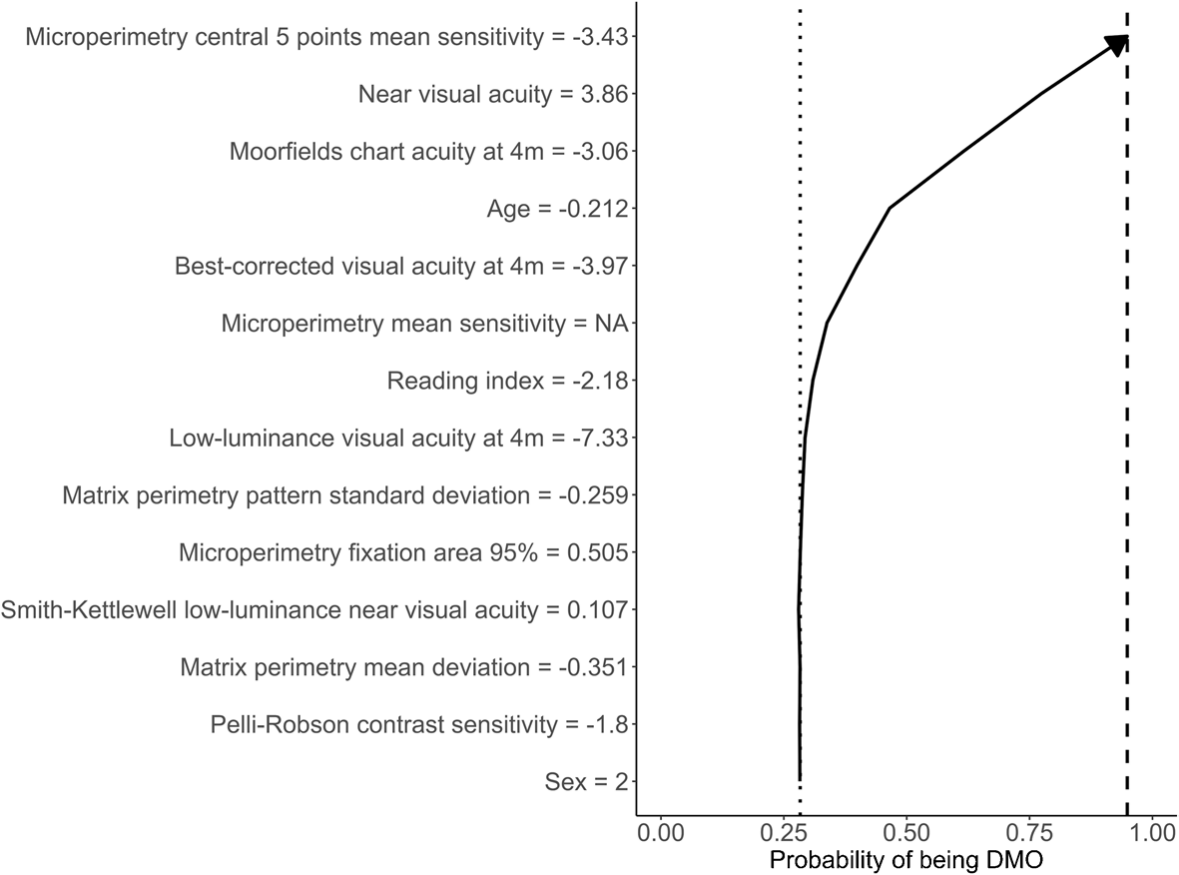
Decision plot showing the influence of variables on prediction for a single eye. Standardised values for each variable are presented after the variable name. The dotted line indicates the overall probability of being DR with DMO in the dataset. The dashed line indicates the predicted probability of being DR with DMO for this eye

Figure 2 illustrates how SHAP values were used to decompose the ensemble model prediction for a single eye for task C. The plot is read from the bottom, with the arrow at each row showing the contribution of that variable (the SHAP value) in moving the predicted probability of being DR with DMO from that expected for the entire dataset (dotted vertical line) to the final prediction for this eye (dashed vertical line). The high predicted probability of DR with DMO was strongly influenced by lower than average values for the microperimetry central 5-point mean sensitivity, NVA, Moorfields chart acuity at 4 m, age and best corrected DVA at 4 m.

The variables with the greatest influence on predictions (ranked by variation in SHAP values) differed by task (Table 3). Microperimetry average sensitivity was the most important for distinguishing the DM no DR group from the no DM group (task A), matrix perimetry pattern standard deviation was the most important for distinguishing the DR no DMO group from the DM no DR group (task B) and microperimetry central 5-point mean sensitivity was the most important for distinguishing the DR with DMO group from the DR no DMO group (task C). These variables contained a high proportion of imputed values, indicating that the ensemble models partially used patterns of imputation to make predictions. Reading index was ranked second for tasks A and B but ninth for task C. NVA was ranked third for task C but was unimportant for the other two tasks. Age ranked fourth or above for all tasks.

**Table 3:** Variable importance by SHAP value standard deviation

Variable	DM no DR vs no DM	DR no DMO vs DM no DR	DR with DMO vs DR no DMO
Microperimetry average sensitivity	0.076 [1] (54.5)	0.023 [13] (55.9)	0.024 [6] (52.0)
Reading index	0.067 [2] (2.3)	0.041 [2] (10.9)	0.015 [9] (10.1)
Microperimetry central 5-point mean sensitivity	0.057 [3] (52.0)	0.026 [8] (53.8)	0.110 [1] (50.3)
Age	0.046 [4] (0.0)	0.038 [4] (0.0)	0.082 [2] (0.0)
Moorfields chart acuity at 4 m	0.037 [5] (24.1)	0.024 [11] (29.4)	0.065 [4] (30.1)
Sex	0.035 [6] (0.0)	0.024 [10] (0.0)	0.000 [14] (0.0)
Matrix perimetry pattern standard deviation	0.032 [7] (72.4)	0.043 [1] (58.1)	0.007 [11] (53.3)
Matrix perimetry mean deviation	0.025 [8] (72.4)	0.025 [9] (58.1)	0.049 [5] (53.3)
Pelli–Robson contrast sensitivity	0.024 [9] (0.4)	0.039 [3] (0.8)	0.001 [13] (0.7)
LLVA at 4 m	0.017 [10] (0.1)	0.021 [14] (0.0)	0.005 [12] (0.0)
Best corrected DVA at 4 m	0.017 [11] (0.0)	0.032 [6] (0.0)	0.023 [7] (0.0)
Microperimetry fixation area 95%	0.016 [12] (52.0)	0.031 [7] (53.8)	0.008 [10] (50.3)
Smith-Kettlewell low-luminance NVA	0.016 [13] (0.2)	0.034 [5] (0.2)	0.021 [8] (0.0)
NVA	0.008 [14] (0.2)	0.023 [12] (0.4)	0.070 [3] (0.0)

Data in square brackets represent importance rank and data in parentheses represent percentage of records imputed

LLVA, low-luminance visual acuity

## Discussion

### Classification using visual function

Ensemble ML models incorporating age, sex and visual function identified the status of diabetic eye disease with high accuracy for three classification tasks. The prominence of tree-based learners in the models indicates that threshold-type associations and interactions among variables were key to accurate prediction. This may explain why ensemble models substantially outperformed the simpler multiple regression models, which lacked the flexibility to model these features. Relying on more conventional regression-based approaches, we would have concluded that visual function measures provide relatively little information with which to distinguish among diabetes mellitus/diabetic retinopathy groups. Approaches based on whether eyes exceed ‘normal ranges’ of variation for single variables are even less likely to have succeeded given the considerable overlap among groups for each of the visual function variables.

Ranking of visual function measures based on SHAP values varied by classification task, indicating that different aspects of visual function are impaired at different stages of diabetic eye disease. Reading index was important for distinguishing between eyes from the no DM, DM no DR and DR no DMO groups. In contrast, NVA and Moorfields chart acuity were important for distinguishing the DR with DMO group from the DR no DMO group. The reading index tests the ability to scan quickly along a line of words, and is therefore sensitive to pathology in both the fovea and parafovea, whereas distinguishing between DR and DMO is achieved best by tests that primarily evaluate foveal function. Microperimetry ranked highly for all tasks, but we hesitate to put too much weight on this finding given the extent of missing data and the use of imputation. Nonetheless our data support the findings of smaller studies that have reported significant changes in microperimetry variables in similar comparisons [34–37].

### Interpretable ML

Interpretable ML enabled us to disentangle associations between visual function and diabetic eye disease and artefacts of the data collection process, at least partially. SHAP values for each eye produced an intuitive visualisation (decision plot) of the variables that most strongly influenced the probability prediction. Clustering eyes by SHAP value enabled us to detect shared patterns of association between visual function variables and diabetic eye disease, highlighting that the same probability prediction of group membership may be made for different reasons, with different visual function variables coming to the fore (see ESM section on interpretable machine learning).

Given the large number of visual function variables and the high proportion of incomplete cases, imputation was necessary to extract the full value from the dataset. Although we used a well-established imputation procedure, ensemble prediction models detected instances when one of the diabetes groups had a larger proportion of missing values for a given variable than the other groups. The proportion of missing values is thus an important consideration when assessing variable importance, especially at the global level, either using variation in SHAP values or other measures of variable importance (e.g. random forest importance). We stress that variable importance in this study and explanations derived from SHAP values are purely phenomenological and do not imply causal links.

Ensemble models detected stratification in the dataset: age ranked highly for all three tasks, reflecting the modest differences in age profile of the four groups, and sex was of moderate importance for task A, for which the sex ratio varied between groups. This reinforces the findings of other studies showing that, to optimise classification accuracy, ML models may focus on statistical artefacts in the data [38, 39].

### Strengths and limitations

A major strength of the study is the number and diversity of visual function variables measured on the same eyes, providing a rich dataset from which to make predictions, and complex enough for ensemble ML to offer substantial improvements over simpler statistical modelling methods. The number of eyes included is also considerably larger than most previous studies of visual function in diabetic eye disease [8, 10, 11, 35–37]. Disease status was determined using ophthalmic image grading for diabetic retinopathy features according to a detailed protocol, providing high-quality labels on which to train models. Diabetic retinopathy status was established using multimodal imaging. The use of ultra-widefield capture on colour images allowed scrutiny of the far retinal periphery where retinopathy may be seen in a proportion of eyes that lack features of diabetic retinopathy elsewhere. The presence of DMO was determined on SD-OCT, which has been shown to be the gold standard for detection of macular features of diabetic retinopathy. In addition, careful identification of the group of patients with diabetes but without retinal manifestations of diabetic retinopathy provided a rare opportunity to characterise visual function changes in early diabetic eye disease. The flexible ensemble ML models made efficient use of the complex structuring of information in the dataset, and combined with the interpretable ML approach, provide a comprehensive framework for modelling using untidy real-world data. Similar approaches have been applied for CVD prediction in diabetes patients [40].

The interpretable ML approach may be applied to any prediction problem in which a trained model is available, and may provide vital checks of performance and integrity when modelling real-world clinical data (e.g. electronic medical records), where informative patterns of missingness and artefacts are present. Interpretability (or explainability) is an important step towards providing trustworthy prediction models [41]. We recognise that richer explanations and frameworks for interpretability and trustworthiness have been emerging [42, 43] and the related concept of actionability has strong appeal [44]. This also serves to remind us that our study and its design have focused essentially on classification tasks, and, given our cross-sectional data, we were not predicting the incidence or progression of disease, for which other SHAP values might imply different actionability, e.g. whether certain visual function tests have more weight or import for predicting retinal disease progression in certain socioeconomic groups and thus merit actions such as altered monitoring or surveillance frequency. By the same token, we must be mindful that data fidelity and representativeness for particular source populations will affect model performance.

Our sample contained few eyes with more advanced diabetic retinopathy (13/216 eyes at grades R2 or R3) so we did not attempt to analyse model performance by diabetic retinopathy subgroup and have limited information on the visual function profile of those with more advanced diabetic retinopathy.

Our study shares limitations common to ML studies, namely risk of overfitting, in which predictive performance is inflated because the model memorises aspects of the training data. We attempted to avoid this by using the SuperLearner algorithm [28, 29], which incorporates a cross-validation stage to avoid overfitting, accounting for interocular correlations within individuals by ensuring that pairs of eyes were not split between training and validation sets. However, in the absence of an external dataset with which to validate our models, it is difficult to assess the extent of any overfitting and the likely performance of our model in a different population or clinical setting.

### Further work

In addition to external validation, a next step towards model application would be to evaluate performance when the number of available visual function measures is constrained. In clinical or trials settings with limited time or funding to assess visual function, it is important to know which combinations of two or three visual function tests perform best for each task, so that tests may be selected appropriately. In many clinical and screening contexts, both imaging and functional tests are available, so designing models to fuse information across modalities is another important step.

### Conclusion

We demonstrated that ML models are capable of classifying the status of diabetic eye disease with moderate to high accuracy using just age, sex and measures of visual function. Use of interpretable ML methods enabled us to identify profiles of visual function associated with diabetic eye disease and to disentangle associations from artefacts of the data collection process. Together, these two techniques have great potential for developing prediction models using real-world clinical data.

### Supplementary Information

Below is the link to the electronic supplementary material.

Supplementary file1 (PDF 366 KB)

## Data Availability

The datasets generated and/or analysed during the current study are available from the corresponding author on reasonable request.

## Abbreviations

DM no DR: Diabetes without diabetic retinopathy (group)
DR no DMO: Diabetic retinopathy without diabetic macular oedema (group)
DR with DMO: Diabetic retinopathy with diabetic macular oedema (group)
DVA: Distance visual acuity
FDT: Frequency doubling technology
ML: Machine learning
NICOLA: Northern Ireland Cohort for the Longitudinal Study of Ageing
No DM: Control group without diabetes
NVA: Near visual acuity
ROC: Receiver operating characteristics
SD-OCT: Spectral domain-optical coherence tomograph
SHAP: SHapley Additive exPlanation
SKILL: Smith-Kettlewell Institute low-luminance
