# Supplementary material for: Identifying the severity of diabetic retinopathy by visual function measures using both traditional statistical methods and interpretable machine learning: a cross-sectional study
Source: Diabetologia. 2023 Sep 19;66(12):2250–60. doi: 10.1007/s00125-023-06005-3 (PMC10627908; doi:10.1007/s00125-023-06005-3)

Identifying the severity of diabetic retinopathy by visual function measures using both traditional statistical methods and interpretable machine learning: a cross-sectional study

David M. Wright[1], Usha Chakravarthy[1], Radha Das[1], Katie W. Graham[1], Timos T. Naskas[1], Jennifer Perais[2], Frank Kee[1], Tunde Peto[1], Ruth E. Hogg[1]

[1]Centre for Public Health, Queen's University Belfast, UK
[2]Wellcome Wolfson Institute for Experimental Medicine, Queen's University Belfast, UK

David M. Wright
d.wright@qub.ac.uk

Electronic Supplementary Material

Interpretable machine learning – clustering

A secondary aim of the analysis was to investigate the ways in which our models handled issues surrounding missing values in the dataset. Specifically, we wanted to know whether the machine learning (ML) models were using blocks of missingness in making predictions and whether these artefacts could be disentangled using interpretable ML techniques. This is a relatively common challenge in datasets of this type and so we included microperimetry and the other variables with substantial missingness (Matrix perimetry and Moorfields chart acuity).

Methods

In addition to the global measures of variable importance based on SHAP values, we used K-means clustering by SHAP values to identify clusters of eyes in which predictions were made for similar reasons. Degree of clustering for each task was determined by visual inspection of elbow plots. We paid particular attention to variation in the proportion of imputed values in each cluster, aiming to identify clusters with the greatest data support as least likely to be influenced by artefacts of imputation.

Results

For task A we identified eight clusters of eyes, two in which eyes were predicted to be DM no DR, but for different reasons in each. For example, the positive SHAP values for reading index, microperimetry central 5 points mean sensitivity, age, and Moorfields chart acuity for cluster Y indicated that these variables contributed most strongly to the prediction of membership in the "DM no DR" group (Figure ESM1). Cluster Y was characterised by below average values for reading index, central 5 points sensitivity and Moorfields chart acuity and above average age (Figure ESM2). In contrast, for cluster V microperimetry average sensitivity contributed most strongly to a "DM no DR" prediction, followed by age and reading index. Cluster V was characterised by above average values for the microperimetry variables and average values for age and reading index. The other tasks highlighted distinct profiles of visual function measurements (clusters of eyes) that produced similar model predictions. In task B eyes in four of eight clusters were predicted to be DR no DMO. In task C, two of five clusters were predicted to be DR with DMO.

Discussion

There was substantial variation in the proportion of imputed values across clusters, indicating that missingness clusters. For example, the global proportion of imputed values for microperimetry for task A was 55%, yet in clusters Y and V, the proportion of missing values for microperimetry was >60% and <50% respectively. Some of the patterns in these clusters are biologically plausible (e.g. below average reading index being associated with increased probability of being DM no DR rather than no DM) so imputation may have mirrored the pattern across the other visual function variables rather than driving the predictions. However, it is impossible to determine whether the imputed values are 'correct', so we interpret predictions from clusters with a larger proportion of missing values with more caution as there is less data support for the patterns detected. In clusters with less imputed values, (e.g. cluster V), more attention should be given to the influential variables with few imputed values such as reading index and Moorfields chart acuity.

Tables

Data sources

Table ESM1. Distribution of eyes by diabetes and retinopathy status and data source.

Data source	No DM	DM no DR	DR no DMO	DR with DMO
NICOLA participant*	1137	158	121	9
Diabetes clinics**	0	120	95	81
Healthy volunteers	180	0	0	0
Total	1317	278	216	90

* Northern Ireland Cohort for the longitudinal study of ageing – prospective cohort study.

** Retinal and metabolic clinics of the Belfast Health and Social Care Trust, Northern Ireland.

Classification using visual function

Table ESM2. Classification performance by model type and diabetes and retinopathy status classification task. TN = True Negative, FN = False Negative, FP = False Positive, TP = True Positive

Task	Model	TN	FN	FP	TP
DM no DR vs No DM	Logistic regression	1311 (82.2%)	256 (16.1%)	6 (0.4%)	22 (1.4%)
DM no DR vs No DM	Ensemble ML	1317 (82.6%)	121 (7.6%)	0 (0.0%)	157 (9.8%)
DR no DMO vs DM no DR	Logistic regression	213 (43.1%)	129 (26.1%)	65 (13.2%)	87 (17.6%)
DR no DMO vs DM no DR	Ensemble ML	278 (56.3%)	2 (0.4%)	0 (0.0%)	214 (43.3%)
DR with DMO vs DR no DMO	Logistic regression	198 (64.7%)	42 (13.7%)	18 (5.9%)	48 (15.7%)
DR with DMO vs DR no DMO	Ensemble ML	208 (68.0%)	42 (13.7%)	8 (2.6%)	48 (15.7%)

Table ESM3 Weighting of learners (estimated meta-learner coefficients) from ensemble fits by diabetes and retinopathy status classification task.

**Variant**	**DM no DR vs No DM**	**DR no DMO vs DM no DR**	**DR with DMO vs DR no DMO**
Intercept only model	0.00	0.00	0.00
LASSO	0.12	0.00	0.64
Ridge regression	0.05	0.32	0.00
Polynomial splines	0.16	0.01	0.00
Random forest	0.45	0.64	0.00
XGBoost - 20 iterations	0.10	0.03	0.00
XGBoost - 50 iterations	0.12	0.00	0.07
Neural network	0.00	0.00	0.00
Bayesian Additive Regression Trees	0.00	0.00	0.29

Figures

Figure ESM1. Influence of variables on classifications for two clusters in which the same classifications were made for different reasons. Positive SHAP values indicate increased probability of being DM no DR.

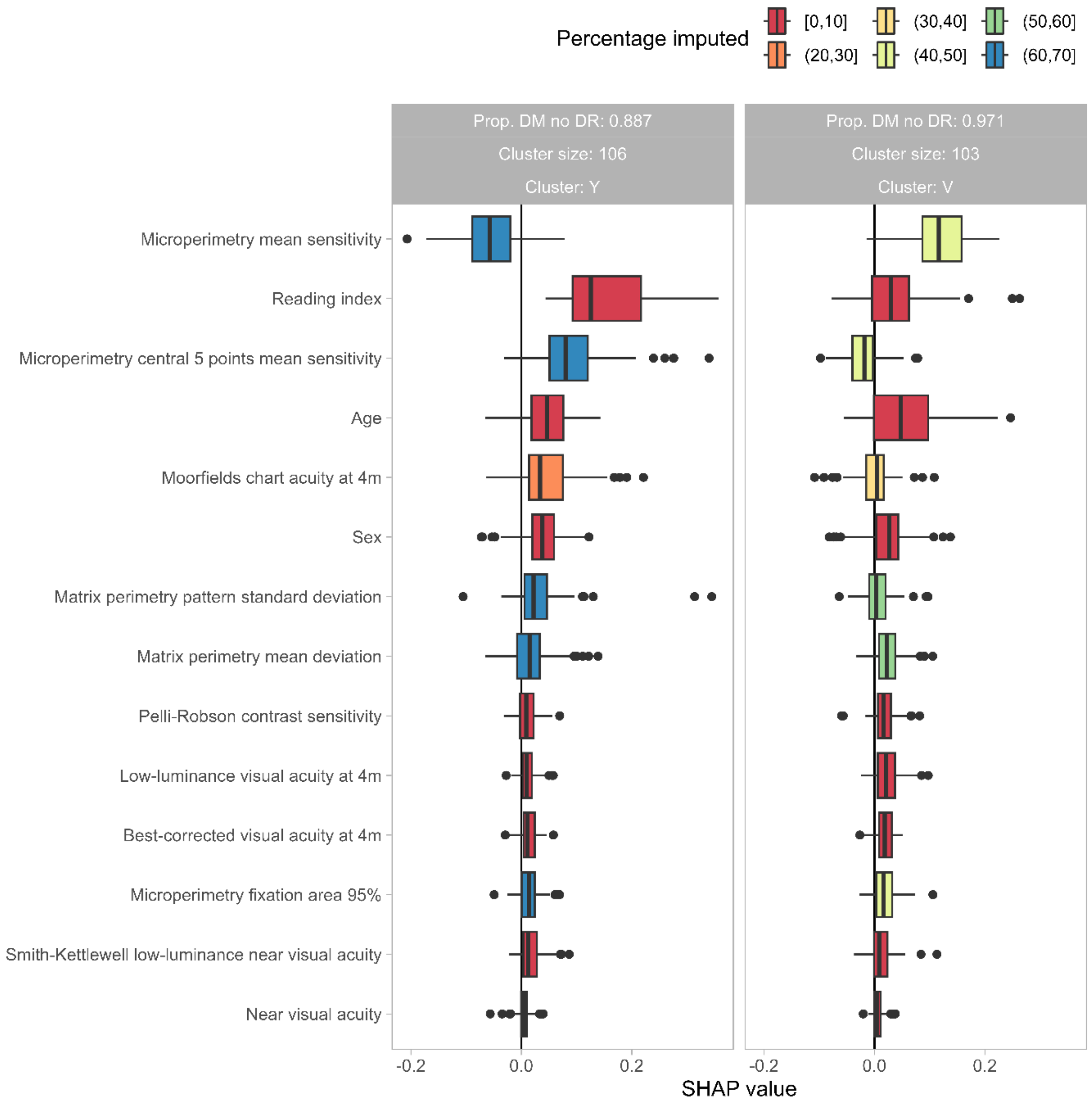

Figure ESM2. Distribution of visual function measurements for two clusters in which the same classifications were made for different reasons.

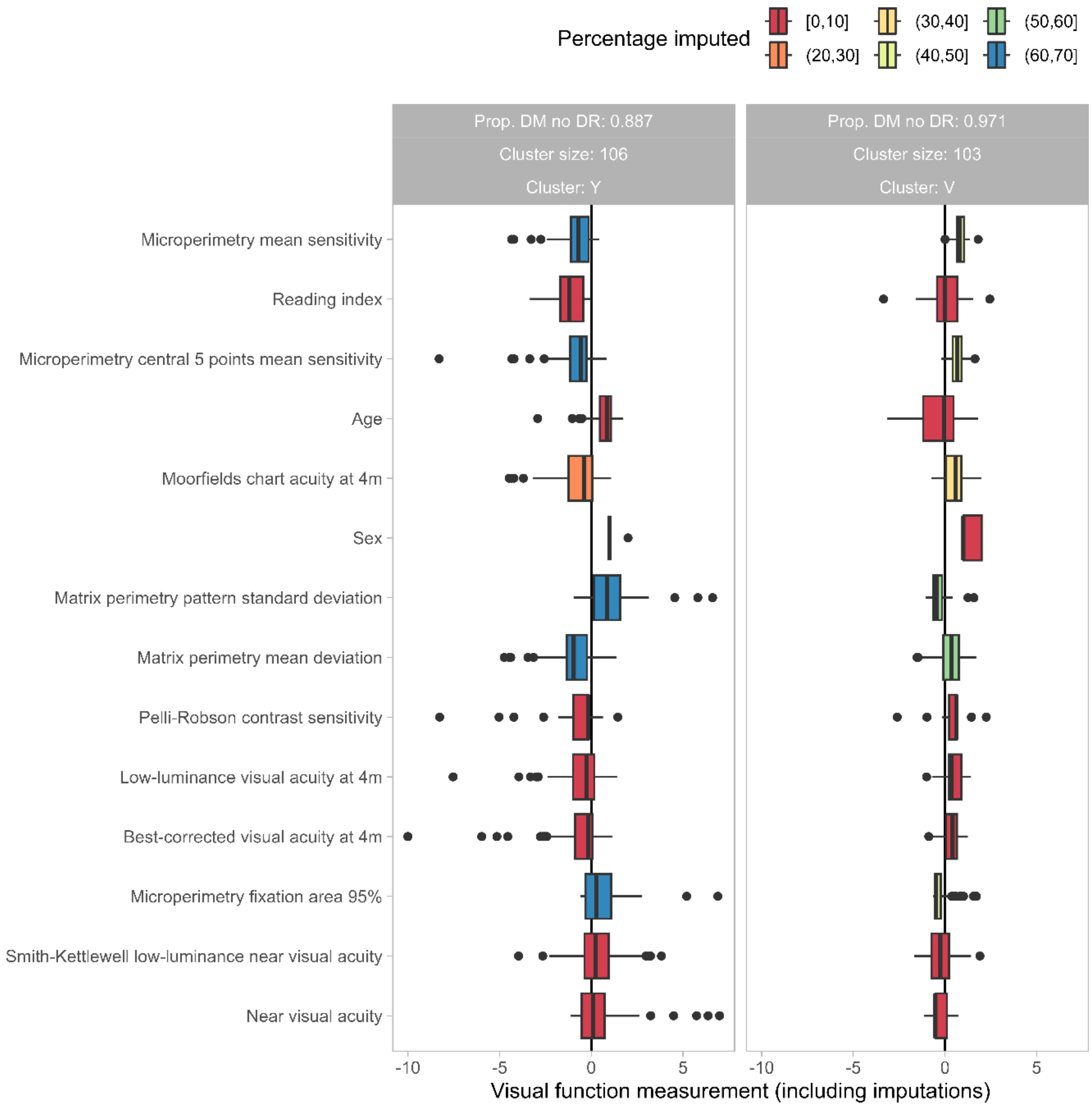

Supplement: Supplementary file 1 — Supplementary file1 (PDF 366 KB) [file 125_2023_6005_MOESM1_ESM.pdf]
